# 

**Published:** 1899-04-15

**Authors:** 


**Notices of Births, Deaths, and Marriages** are inserted in this column at a charge of 2s. 6d. for an announcement not exceeding four lines.

### NOTICE TO CORRESPONDENTS.

All MS., Letters, Books for Review, and other matters intended for the Editor should be addressed The Editor, "The Hospital," The Hospital Building, 28 & 29, Southampton Street, Strand, W.C.
The Editor cannot undertake to return rejected MS., even when accompanied by stamped directed envelope.

**Notice to Advertisers and Subscribers.**

All Advertisements, Orders for Copies of The Hospital, and Business Communications should be addressed to **THE MANAGER (Not to the Editor)**, The Hospital, The Hospital Building, 28 & 29, Southampton Street, Strand, London, W.C.
The Cost of Subscription, post free, is as follows:—Per week, 2½d.; per quarter, 2s. 8d.; per half-year, 5s. 3d.; per annum, 10s. 6d. Foreign:—Per annum, 15s. 2d., payable, in all cases, in advance.

**NOTICE.—Vol. XXV. of "The Hospital" (October, 1898, to March, 1899), in handsome cloth binding, will shortly be on sale at the Office of this Journal, price 7s. 6d. Cases for binding the Numbers contained in this Volume 1s. 6d. Index to Vol. XXV., 2½d., post free.**

**The Monthly Part for April is now ready. Price 6d., by post 8d.**

### BOOKS RECEIVED.

Baillière, Tindall, and Cox.

"The Chemical and Biological Examination of Water." By T. H. Pearmain and C. G. Moor, M.A.

"The Administrative Control of Tuberculosis." By Sir Richard Thorne Thorn, K.C.B., F.R.S. Being the Harben Lectures delivered in 1898.

The "Lancet" Office.

"Disease: Its Treatment and the Medical Profession in 1899." By William Ewart, M.D., F.R.C.P. The Harveian Lectures, reprinted from the *Lancet*.

Macmillan and Co.

"A System of Medicine." Edited by Professor Thomas Clifford Allbutt, M.D., F.R.C.P., F.R.S. Volume VI.

John Wright and Co.

"The Medical Annual and Practitioners' Index. 1899."

Swann Sonnenschein and Co.

"Text-Book of the Embryology of Invertebrates." By Dr. E. Korschelt and Dr. K. Heider. Translated by Matilda Bernard. Edited by Martin F. Woodward.

W. H. and L. Collingridge.

"The City of London Directory, 1899."

### Scraps and Gleanings.

An attempt is being made to inaugurate a movement in aid of a cottage hospital for Avonmouth.

At the Sydney Hospital, New South Wales, 116 patients were treated in 1898, as compared with 51 the previous year.

The new hospital which is being built in Rangoon by the Dufferin Fund will be opened this month. It will cost Rs.72,000.

By the time the new works at the Royal South Hants Infirmary are completed Mr. Garton's generous gift will have amounted to over £7,000.

A Ladies' Committee in connexion with the Belfast Samaritan Hospital has lately been formed, and fourteen ladies therefrom are to form an executive.

The endowment fund of the Cornelia Hospital, Poole, amounts now to £2,249 4s. 3d. The subscriptions have shown a falling off, barely one-fourth of the required sum having been subscribed.

It has been decided to enlarge and improve the Victoria Hospital, Winchester, by the addition of an administrative block, with living and sleeping rooms for eight nurses, and other accommodation.

New plans for the proposed Spennymoor Infectious Hospital have been prepared and forwarded to the Local Government Board. It has been found necessary to recommend that a loan of £4,000 should be applied for.

### The Student of Medicine.

### APPOINTMENTS.

A. J. Anderson, M.B., C.M., appointed Medical Officer of the Staines Union Workhouse. E. G. E. Arnold, M.R.C.S.Eng., L.R.C.P.Lond., appointed Senior Assistant Medical Officer of the Toxteth Park Workhouse. A. H. Back, M.A.Camb., L.R.C.P.Edin., M.R.C.S.Eng., appointed Medical Officer for the Acle District of the Blofield Union. E. Barrett, L.R.C.P.Lond., M.R.C.S.Eng., appointed Medical Officer for the Maxey District of the Peterborough Union. P. T. B. Beale, F.R.C.S.Eng., appointed Surgeon to In-patients at the Great Northern Central Hospital. J. Brownlee, M.A., M.D.Glasg., D.P.H.Camb., appointed Medical Officer of Health for the Island of Guernsey. G. B. Buttery, L.R.C.P.Edin., L.F.P.S.Glasg., appointed Medical Officer of Health for the Oldbury Urban District. B. R. C. Christie, M.B., C.M.Edin., appointed a Resident House Physician to the Glasgow Royal Infirmary. W. R. Dawson, M.D.&c.Dubl., M.R.C.P.I., to be Medical Superintendent, Farnham House Asylum, Finglas, Dublin. A. Dunbar, M.B., C.M.Edin., appointed Medical Officer for the Acomb District of the Great Ouseburn Union. A. E. Horn, B.Sc.Lond., M.R.C.S., L.R.C.P., appointed Assistant Medical Officer to the Chelsea Infirmary. S. Hosegood, M.R.C.S.Eng., L.S.A., appointed Medical Officer for the Swinton and Clifton Districts of the Barton-upon-Irwell Union. T. N. Kelynack, M.D.Vict., M.R.C.P.Lond., appointed Honorary Pathologist to the Manchester Clinical Hospital for Women and Children. W. Liversidge, M.B.Lond., M.R.C.S.Eng., L.R.C.P.Lond., appointed Senior House Surgeon at the Blackburn and East Lancashire Infirmary. J. F. McClean, M.R.C.S., L.R.C.P., appointed Surgeon Superintendent of the British Hospital, Constantinople. W. H. Parry, M.B., C.M.Glag., appointed Medical Officer for the Workhouse and the Bettws-y-Coed and Trefriw District of the Llanrwst Union. F. B. Rutter, M.D.Durh., F.R.C.S.Eng., appointed Medical Officer of the Workhouse and the First District of the Mere Union.

